# Comparison Between Nailing and Plating in the Treatment of Distal Tibial Fractures: A Meta-Analysis

**DOI:** 10.1177/1457496920957830

**Published:** 2020-09-14

**Authors:** E. Ekman, K. Lehtimäki, J. Syvänen, M. Saltychev

**Affiliations:** 1 Department of Orthopedics and Traumatology, Turku University Hospital, Turku, Finland; 2 Department of Pediatric Orthopedics, Turku University Hospital, Turku, Finland; 3 Department of Physical and Rehabilitation Medicine, Turku University Hospital, University of Turku, Turku, Finland

**Keywords:** Distal tibial fracture, meta-analysis, plate, nail, functional outcomes, complications

## Abstract

**Background and Aims:**

To evaluate evidence on the superiority of plate fixation over intramedullary nail fixation in the treatment of distal tibial fractures regarding functional outcomes and complication rates.

**Material and Methods:**

Cochrane Controlled Trials Register, Medline, Embase, CINAHL, Scopus, and Web of Science databases were searched in December 2019. The risk of systematic bias was assessed according to the Cochrane Collaboration’s domain-based evaluation framework.

**Results:**

The search resulted in 514 records, the final sample included 10 randomized controlled trials (782 patients). There were statistically significant differences in operating time (−11.2, 95% confidence interval: −16.3 to −6.1 min), time to partial weight bearing (−0.96, 95% confidence interval: −1.8 to −0.1 weeks), time to full weight bearing (−2.2, 95% confidence interval: −4.32 to −0.01 weeks), the rates of deep infections (risk ratio = 0.37, 95% confidence interval: 0.19 to 0.69), and the rates of soft-tissue complications (risk ratio = 0.52, 95% confidence interval: 0.33 to 0.82) favoring intramedullary nail. Intraoperative blood loss (127.2, 95% confidence interval: 34.7 to 219.7 mL) and postoperative knee pain and stiffness (relative risk = 5.6, 95% confidence interval: 1.4–22.6) showed significant differences favoring plate fixation. When combining all complication rates, the difference was risk ratio = 0.77 (95% confidence interval: 0.63 to 0.95) favoring intramedullary nail. No significant differences in radiation time, length of incision, length of hospital stay, time to return to work, time to union, the rates of healing complications or secondary procedures, ankle pain or stiffness, or functional scores were found.

**Conclusion:**

This meta-analysis suggests that intramedullary nail might be slightly superior in reducing postoperative complications and result in slightly faster healing when compared to plate fixation.

## Introduction

Fractures of the distal tibia are relatively rare, with a reported annual incidence of 9.1 per 100,000. These fractures can occur in both high-energy and low-energy trauma, while simple falls are the most common mechanism of injury (1). Distal tibial fractures are almost always treated surgically because conservative treatment involves long leg casts, prolonged immobilization, and a high risk of malunion (2, 3). Only very comorbid patients who are not likely to tolerate anesthesia and patients with non-displaced fractures are treated conservatively (4).

The distal tibia is defined according to Müller and AO/Association for the Study of Internal Fixation (ASIF) definitions as a fracture primarily located within the “Müller square,” which is a square with sides of a length defined by the widest portion of the tibial plafond (5, 6). In practice, distal tibial fractures include the more proximal metaphysis and distal diaphysis (lower third of the tibia). Fractures with a simple extension of a non-displaced fracture line into the ankle joint are treated in a manner similar to extra-articular fractures.

The most commonly used surgical techniques are intramedullary nailing (IMN) and plate fixation (PF). PF is performed by using either open reduction and internal fixation (ORIF) or minimally invasive percutaneous osteosynthesis (MIPO). IMN is minimally invasive with the benefit of small skin incisions, minimal soft-tissue trauma, and preservation of extraosseous blood supply (5). The fixation is stable and allows early mobilization (7). However, anterior knee pain is possible and malunions have been reported with distal tibial fractures (8, 9). ORIF has a low risk of malunion, but longer time to weight bearing and increased risk of wound complications (10). However, MIPO results in less damage to the blood supply of the distal tibia and hence reduces the risk of wound complications (11).

The purpose of our study is to evaluate evidence on the superiority of PF over IMN fixation in the treatment of distal tibial fractures regarding functional outcomes and complication rates.

## Material and Methods

### Inclusion and Exclusion Criteria (PICO)

The criteria for considering studies for this review were based on the Population, Intervention, Comparison, and Outcome (PICO) framework as follows:

- Patients: Adults (>16 years) with fractures in distal tibia (fractures extending within 2 Müller squares (approximately 11 cm) from the tibial plafond and fractures with no or with simple extension of a non-displaced fracture line into the ankle joint) caused by trauma. Other fractures related to osteoporosis, tumors, and systemic diseases were excluded (according to the original articles inclusion and exclusion criteria).
- Papers: Randomized controlled trials (RCTs) published in English in peer-reviewed academic journals. Abstracts available. Excluding theses, conference proceedings, case reports, and pilot reports.
- Databases: Medline via PubMed, Embase, Scopus, Web of Science, Central.
- Intervention and comparison: Intramedullary nailing versus plate osteosynthesis.
- Outcome: Difference between groups in functional outcomes and complication rates.

The search clause at Medline was as follows:

((“Tibia/injuries” [Mesh] OR “Tibia/surgery” [Mesh] OR “Tibia/therapy” [Mesh]) OR tibia*[TIAB]” [Mesh] OR fractur* [TIAB]) NOT (pediatr*[TI] OR paediatr*[TI] OR review[TI] OR meta-analy*[TI] OR protocol*[TI] OR pilot*[TI])

In order to avoid missing potentially relevant studies, the use of other limiters and filters was restricted, and the authors relied instead on manual selection. Similar clauses were used when searching the other databases. The references of identified articles and reviews were also checked for relevance.

### Selection Strategy

**Fig. 1.**
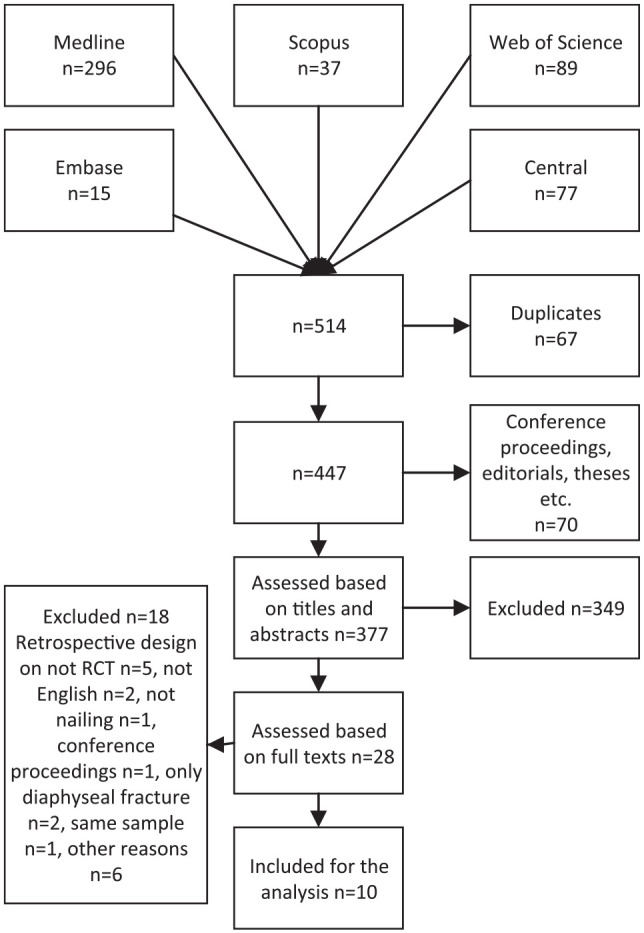
Search flow.

The records identified from the data sources were stored using Endnote software (Endnote X7.8, Thomson Reuters). Using a built-in search engine for the Endnote software, duplicates, conference proceedings, theses, reviews, and case reports were deleted. Two independent reviewers screened the titles and abstracts of the remaining articles and assessed the full texts of potentially relevant papers (Fig. 1). Disagreements between the reviewers were resolved by consensus or by a third reviewer.

### Extraction Strategy

The data needed for a quantitative assessment were extracted using a standardized form based on recommendations by the Cochrane Handbook for Systematic Reviews of Interventions (12). The form included the first author name, the year of publication, country, group sizes at randomization and the end of follow-up, gender distribution, the average age of patients within groups, inclusion and exclusion criteria, main conclusions, and the estimates of main outcomes.

### Assessment of the Methodological Risks of Systematic Bias

Two independent reviewers rated the methodological quality of the included trials using the Cochrane domain-based quality assessment tool (12). Each study was rated as having “low,” ‘high,’ or “unclear” risk of systematic bias in seven domains. Domains were assessed in the following sequence: (1) selection bias (randomized sequence generation and allocation concealment), (2) allocation concealment, (3) performance bias (blinding of participants and personnel), (4) detection bias (blinding of outcome assessment), and (5) attrition bias (incomplete outcome data—e.g. due to dropouts), (6) reporting bias (selective reporting), and (7) other sources of bias. Disagreements between the reviewers were resolved by consensus or by a third reviewer.

## Statistics

### Statistical Model, Heterogeneity, and Publication Bias

A random-effects model was used to quantify the pooled effect size of the included studies, which was a more fitting choice than a fixed-effect model considering the context of medical decision-making and generalizing the results beyond the selected samples. The results were accompanied by 95% confidence intervals (95% CIs) and a two-tailed *p* value (significant if ⩽0.05) when appropriate. In some cases, the variances were adopted from reported 95% CIs. The test for heterogeneity was conducted using the Q test; heterogeneity was deemed present if Q was greater than the degree of freedom (number of studies – 1). The I² statistic describes the percentage of variability in effect estimates due to heterogeneity rather than sampling error (chance). Publication bias was not assessed as the number of studies in the model was <10.

### Outcome Measures Employed in the Meta-Analysis

Functional scales used by the included studies varied. Thus, when pooling functional scores, the estimate was presented as a standardized mean difference (SMD). In that case, the effect size was considered small when SMD was 0.2, medium when SMD was 0.5, and large when SMD was 0.8 or higher. Other continuous variables were pooled as a raw mean difference. The complication rates were pooled using a risk ratio (RR). The definitions of complications varied widely across the studies. In order to pool the reported estimates, the reported complications were assigned to one of the seven groups: ankle pain or stiffness, deep infections, soft-tissue complications (delayed wound healing, superficial infection, or soft-tissue irritation), knee pain or stiffness, healing complications (delayed union, malunion, or non-union), and secondary procedures. Some reported complications were disregarded, as they were reported by single studies and there was no certainty of whether they were complications at all (e.g. the use of pain medications). Some outcomes were disregarded, as the variance estimates were not reported.

All calculations for the meta-analysis were performed using Comprehensive Meta-Analysis CMA software, Version 3.0, available from www.meta-analysis.com and Microsoft^®^ Excel^®^ 2016.

## Results

The search resulted in 514 articles (Fig. 1). After screening, 10 RCTs were considered relevant (13–22). One study reported outcomes at 6 months and at 1 year in two different publications (22, 23). In that case, the latter paper with 1-year results was included (22).

In total, the included RCTs involved 782 patients: 392 treated with IMN and 390 with plating. The sample sizes varied from 24 to 314 (nailing and plating together). The demographic characteristics of the included studies are summarized in Table 1. In only one study, patients with simple extension of a non-displaced fracture line into the ankle joint were included (22). All studies used minimally invasive locking plate (MIPO) fixation and reamed interlocking nail fixation. In all studies, the plate was positioned medially on the distal tibia. In the IMN group, the patella tendon splitting incision was used in four studies (15, 16, 18, 19); in one study, the patella tendon was retracted laterally (20), and five studies did not specify the used incision (13, 14, 17, 21, 22). A postoperative short-leg splint or cast was used for 3 weeks in one study (13); five studies allowed active range of motion in ankle and knee joints during the first postoperative week (14, 16, 18, 20, 22), while the rest did not specify the immobilization protocol (15, 17, 19, 21). In all studies, the allowance of weight bearing was assessed individually, usually only after a bony callus was seen in X-ray images. The functional scores used in the studies were: The American Orthopedic Foot and Ankle Surgery (AOFAS) scoring system, Disability Rating Index (DRI), Olerud and Molander Ankle Score (OMAS), The EuroQol EQ-5D generalized health outcome questionnaire, Foot Function Index (FFI), Musculoskeletal Function Assessment (MFA), and Teeny and Wiss Clinical Assessment Criteria. The patient follow-up varied from 9 to 71 months. The overall systematic risk of bias was considered low in 2 (14, 22) and high in 8 (13, 15–21) of the included RCTs (Table 2).

**Table 1:** Demographic characteristics of the included studies.

	Country	Follow-up	Number of patients		Women		Mean age		AO classification	Fracture type
			Nail	Plate	Nail	Plate	Nail	Plate		
Guo et al. (13)	China	1 year	44	41	18%	17%	44	44	43A	Closed, type I
Mauffrey et al. (14)	UK	1 year	12	12	42%	25%	50	33	43A	Closed, type I
Vallier et al. (15)	USA	1 year	45	41	18%	12%	41	38	42A, B, C	Closed, type I, II,
Pawar et al. (16)	India	1 year	15	15	10%^a^		42^a^		43A	Closed
Polat et al. (17)	Turkey	1 year	10	15	10%	53%	34	36	42A	Closed
Fang et al. (18)	China	2 years	28	28	32%	25%	35	39	42A, B, C	Closed, type I, II
Ali et al. (19)	India	9 months	30	30	23%	30%	42	40	42A, B, C, 43A	Close, type I
Daolagupu et al. (20)	India	1 year	21	21	19%	29%	35	39	43A	Closed
Wani et al. (21)	India	1 year	30	30	27%	33%	36	38	42A, B, C	Closed
Costa et al. (22)	UK	1 year	136^b^ 157^c^	116^b^ 157^c^	40%	37%	44	46	Fracture extending within 2 Müller squares of the ankle joint	Closed

a Entire sample.

b Sample size for pre-treatment analysis (approximated).

c Sample size for reported complications.

**Table 2:** Risk of systematic bias of the included studies.

	Random sequence generation (selection bias)	Allocation concealment (selection bias)	Blinding of participants and personnel (performance bias)	Blinding of outcome measurement (detection bias)	Incomplete outcome data (attrition bias)	Selective reporting (reporting bias)	Other	Overall risk of systematic bias
Guo et al. (13)	Unclear	Unclear	High	High	High	High	High	High
Mauffrey et al. (14)	Low	Low	High	Low	High	Low	Low	Low
Vallier et al. (15)	Low	Low	High	High	High	Low	Unclear	High
Pawar et al. (16)	High	High	High	High	High	High	High	High
Polat et al. (17)	Low	Unclear	High	High	Low	High	Unclear	High
Fang et al. (18)	Low	Low	Unclear	Unclear	Low	High	High	High
Ali et al. (19)	High	High	High	Unclear	High	High	High	High
Wani et al. (21)	Low	Unclear	High	High	High	High	High	High
Daolagupu et al. (20)	Low	Low	High	High	Unclear	High	High	High
Costa et al. (22)	Low	Low	High	Unclear	Low	Low	Low	Low

**Fig. 2.**
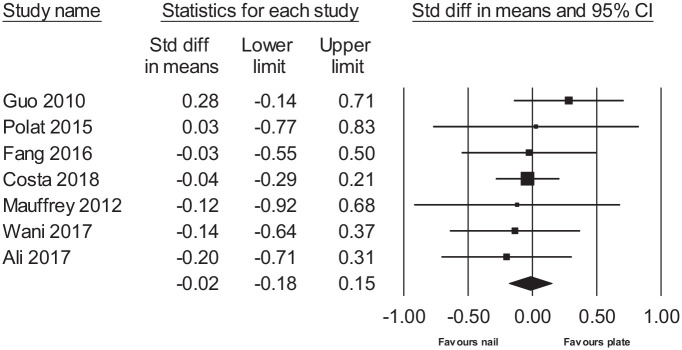
Forest plot of functional scores.

**Fig. 3.**
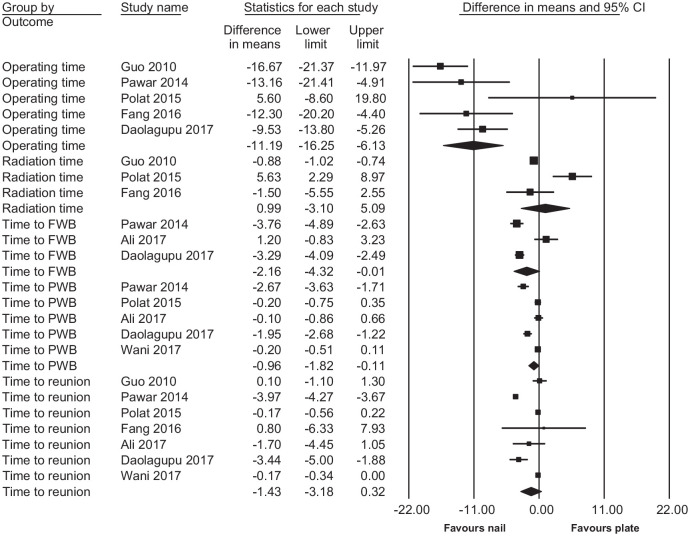
Forest plot of pooled continuous outcomes (raw mean difference).

There was no statistically significant difference in radiation time during surgery (raw MD = 0.99, 95% CI: −3.10 to 5.09 min), length of incision (raw MD = 1.00, 95% CI: −0.21 to 2.21 cm), length of hospital stay (raw MD = 0.10, 95% CI: −3.11 to 3.31 days), time to return to work (raw MD = 0.30, 95% CI: −6.13 to 6.73 weeks), time to union (raw MD = −1.43, 95% CI: −3.18 to 0.32 weeks), healing complications (RR = 1.26, 95% CI: 0.85 to 1.87), secondary procedures (RR = 0.78, 95% CI: 0.51 to 1.20), ankle pain or stiffness (RR = 0.72, 95% CI: 0.34 to 1.54), or others (RR = 0.63, 95% CI: 0.31 to 1.25). Also, during the follow-up (scores measured varying from 3 to 12 months), no difference was found in the change in functional scores (AOFAS, DRI, OMAS, EuroQol EQ-5D, FFI, MFA, and Teeny and Wiss Clinical Assessment Criteria) (SMD = −0.02, 95% CI: −0.18 to 0.15) between the two groups (Fig. 2). There was a statistically significant difference in operating time (raw MD = −11.19, 95% CI: −16.25 to −6.13 min), time to partial weight bearing (PWB) (raw MD = −0.96, 95% CI: −1.82 to −0.11 weeks), time to full weight bearing (FWB) (raw MD = −2.16, 95% CI: −4.32 to −0.01 weeks), deep infection (RR = 0.37, 95% CI: 0.19 to 0.69), and soft-tissue complications (RR = 0.52, 95% CI: 0.33 to 0.82), favoring IMN. In intraoperative blood loss (raw MD = 127.20, 95% CI: 34.73 to 219.67 mL) and postoperative knee pain and stiffness (RR = 5.64, 95% CI: 1.41 to 22.58), there was a statistically significant difference favoring PF. When combining all analyzed postoperative complication rates, the difference was (RR = 0.77, 95% CI: 0.63 to 0.95) favoring IMN. The results are presented in Table 3 and forest plots for continuous outcomes in Fig. 3.

**Table 3:** Pooled estimates.

Outcome	Number of studies	Estimate	95% CI		Q	df	I^2^
			Lower	Upper			
Risk ratios (RR) of complications—RR < 1.0 favors nailing							
Ankle pain or stiffness	4	0.72	0.34	1.54	4.34	3	31
Deep infection	5	0.37	0.19	0.69	0.19	4	0
Soft-tissue complications	9	0.52	0.33	0.82	7.03	8	0
Knee pain or stiffness	5	5.64	1.41	22.58	7.94	4	50
Healing complications	8	1.26	0.85	1.87	2.74	7	0
Others	5	0.63	0.31	1.25	4.42	4	9
Secondary procedures	5	0.78	0.51	1.20	2.10	4	0
All together	n/a	0.77	0.63	0.95	53.57	40	25
Continuous outcomes—raw mean difference—<0 favors nailing							
Operating time	5	−11.19	−16.25	−6.13	11.06	4	64
Radiation time	3	0.99	−3.10	5.09	14.67	2	86
Time to FWB	3	−2.16	−4.32	−0.01	18.73	2	89
Time to PWB	5	−0.96	−1.82	−0.11	40.33	4	90
Time to union	7	−1.43	−3.18	0.32	494.51	6	99
Blood loss	1	127.20	34.73	219.67	0.00	0	0
Time to return to work	1	0.30	−6.13	6.73	0.00	0	0
Hospital stay	1	0.10	−3.11	3.31	0.00	0	0
Length of incision	1	1.00	−0.21	2.21	0.00	0	0
Continuous outcomes—standardized mean difference—<0 favors nailing							
Functional score	7	−0.02	−0.18	0.15	2.70	6	0

CI: confidence interval; RR: risk ratios; FWB: full weight bearing; PWB: partial weight bearing.

## Discussion

The aim of this meta-analysis was to compare the efficacy and complications of plate and nail fixation in the treatment of distal tibial fractures. In the IMN group, the length of operation was statistically significantly shorter, as was the time to partial and full weight bearing, and there were statistically significantly fewer deep infections, soft-tissue complications, and postoperative complications all together. The PF group demonstrated less intraoperative blood loss and postoperative knee pain and stiffness. Regarding other parameters, there were no statistically significant differences.

Previously, several meta-analyses have been published comparing IMN and PF in the treatment of distal tibial fractures (24–28), but only two of them included solely RCTs (27, 28). We only included RCTs with functional results, leading to inclusion of partially different studies than previous meta-analysis. In the patient’s perspective, the functional outcome is as important as avoiding complications. We also identified two RCTs (16, 19) that were not included in the meta-analysis by Guo et al. (27) and Hu et al. (28) and a longer follow-up for the study by Costa et al. (22). These differences in the included studies led to in part different results.

There were statistically significantly more deep infections and other wound problems (delayed wound healing, superficial infection, and soft-tissue irritation) when the plate was used. The distal tibia has medially delicate blood supply and thin soft-tissue coverage, making this area vulnerable to wound problems, which might explain these between-group differences (11). Regarding other wound problems, our findings are in line with previous meta-analyses (25, 26, 27, 28). However, regarding deep infections, only Guo et al. (27) reported the same result. This can be partly explained by differences in the patient populations. Hu et al. (28) included a study in which the mean age of the patients in the plating group was significantly lower than the mean age of the patients in the nailing group (29). This study comprised 21% of the meta-analysis’ plating group. Younger patients are in general healthier and have better wound healing capacity than older patients and this might have an impact on deep infection rates.

Weight bearing was allowed earlier for patients with IMN than for patients treated with PF, indicating that calluses were seen earlier on X-rays in the IMN group. Also, IMN tolerates axial loading better than PF, which might guide individual assessment toward earlier weight bearing in the IMN group (7). However, this finding did not translate into a quicker return to work, time to union, or better functional outcome. Time to weight bearing was not studied in earlier meta-analyses.

Shorter operating time in the IMN group is likely explained by the use of a traction table, which makes reduction quicker and easier to maintain than techniques used in MIPO (13). Of the previous meta-analysis, one had the same finding (27), while another found no difference (28). Previous evidence of radiation time is conflicting (27, 28); however, we found no difference between the groups.

We found no difference in malunion, delayed union, non-union, or time to union. This is consistent with previous meta-analysis (25, 27, 28). However, one meta-analysis reported a higher risk of malunion in the IMN group (28). This meta-analysis includes a larger proportion of older studies, and the result might be due to variations in nail design and operative techniques over time, making multiple screw insertions more distally possible, thus increasing mechanical stability (30).

The results are consistent with previous meta-analyses in that IMN has a greater risk of knee pain and stiffness (25, 27, 28). This can be explained by the operative technique of IMN through the patella tendon, which is generally regarded as the cause of anterior knee pain (31). We found no difference in functional scores, which is also consistent with previous meta-analysis (25, 27, 28). No difference in secondary procedures was detected either, confirming the previous findings (25).

There are several limitations in this study. First, the majority of the included RCTs were prone to risk of systematic biases and were conducted on relatively small samples. Second, there was a diversity in used evaluation scales. Third, only half the included RCTs described the incision used for IMN, which might be important regarding postoperative knee pain and stiffness. Also, the inclusion and exclusion criteria varied.

Both PF and IMN are viable options in the treatment of distal tibial fractures. However, our data suggest that in high-risk patients, IMN is a safer choice as it has a lower risk of complications. However, for some patients with low risk of infection and soft-tissue complications avoiding postoperative knee pain and stiffness might be more important. In the future, high quality studies are needed to identify patient and fracture-related factors that might guide the choice of fixation method individually leading to the least complications and best possible function. Also, a meta-analysis of the treatment of distal tibial fractures comparing PF and IMN performed via the suprapatellar approach should be conducted to investigate whether this has an effect on between-group differences in postoperative knee pain and stiffness.

In conclusion, this meta-analysis suggests that IMN might be slightly superior in reducing postoperative complications when compared to PF, but there was no difference in functional outcomes. The IMN may also result in slightly faster healing.
